# Replication-Independent Endogenous DNA Double-Strand Breaks in *Saccharomyces cerevisiae* Model

**DOI:** 10.1371/journal.pone.0072706

**Published:** 2013-08-19

**Authors:** Jirapan Thongsroy, Oranart Matangkasombut, Araya Thongnak, Prakasit Rattanatanyong, Siwanon Jirawatnotai, Apiwat Mutirangura

**Affiliations:** 1 Inter-Department Program of BioMedical Sciences, Faculty of Graduate School, Chulalongkorn University, Bangkok, Thailand; 2 Department of Microbiology and Developing Research Unit on Oral Microbiology, Faculty of Dentistry, Chulalongkorn University, Bangkok, Thailand; 3 Department of Anatomy, Faculty of Medicine, Chulalongkorn University, Bangkok, Thailand; 4 Center for Excellence in Molecular Genetics of Cancer and Human Diseases, Chulalongkorn University, Bangkok, Thailand; 5 Department of Cancer Biology, Dana-Farber Cancer Institute, and Department of Genetics, Harvard Medical School, Boston, Massachusetts, United States of America; 6 Department of Pharmacology, Faculty of Medicine Siriraj Hospital, Mahidol University, Bangkok, Thailand; Universita' di Milano, Italy

## Abstract

Without exposure to any DNA-damaging agents, non-dividing eukaryotic cells carry endogenous DNA double-strand breaks (EDSBs), or Replication-Independent (RIND)-EDSBs. In human cells, RIND-EDSBs are enriched in the methylated heterochromatic areas of the genome and are repaired by an ATM-dependent non-homologous end-joining pathway (NHEJ). Here, we showed that *Saccharomyces cerevisiae* similarly possess RIND-EDSBs. Various levels of EDSBs were detected during different phases of the cell cycle, including G0. Using a collection of mutant yeast strains, we investigated various DNA metabolic and DNA repair pathways that might be involved in the maintenance of RIND-EDSB levels. We found that the RIND-EDSB levels increased significantly in yeast strains lacking proteins involved in NHEJ DNA repair and in suppression of heterochromatin formation. RIND-EDSB levels were also upregulated when genes encoding histone deacetylase, endonucleases, topoisomerase, and DNA repair regulators were deleted. In contrast, RIND-EDSB levels were downregulated in the mutants that lack chromatin-condensing proteins, such as the high-mobility group box proteins, and Sir2. Likewise, RIND-EDSB levels were also decreased in human cells lacking HMGB1. Therefore, we conclude that the genomic levels of RIND-EDSBs are evolutionally conserved, dynamically regulated, and may be influenced by genome topology, chromatin structure, and the efficiency of DNA repair systems.

## Introduction

Endogenous DNA double strand breaks (EDSBs) can occur spontaneously without any exogenous insults [1]. EDSBs are generally believed to result from a variety of events, such as DNA replication through single stranded lesions and mechanical stress [2]. Previous studies in human cells lacking genes involved in DSB repair showed that EDSBs could arise as often as 50 times per cell cycle, but most are rapidly repaired in normal cells [1]. Although the majority of spontaneous DSBs are efficiently repaired, inaccurate repair of EDSBs could be a cause of carcinogenic mutations [1]. Therefore, in normal cells, there should exist mechanisms to avoid error-prone repair of EDSBs that could protect the genome from potentially hazardous mutations or rearrangements [3], [4].

Recently, we developed a new technique to detect EDSBs based on Interspersed Repetitive Sequence Ligation-Mediated PCR (IRS-LMPCR) [3]. Linker oligonucleotides were ligated to existing DNA ends in the genome, and the EDSBs were measured by PCR using primers specific to the linker and the IRSs that widely distribute throughout the genome. This method is more sensitive than the comet assay [5] and does not rely on H2AX phosphorylation [6]. Using this technique, we detected EDSBs in proximity to IRSs in several human cell lines in the absence of any DNA damage inducer [3].

Intriguingly, we found that during the G0 phase, human cells possess a significant number of IRS-EDSBs. Because these breaks are specific to the non-replicative stage of the cells, we termed them “Replication-INDependent EDSBs” (RIND-EDSBs) [4]. Under normal physiological conditions, RIND-EDSBs are hypermethylated, localized within facultative heterochromatin, devoid of γH2AX, and repaired by the ATM-dependent non-homologous end-joining pathway (NHEJ) [4]. We further showed a rapid increase in γH2AX and a reduction of RIND-EDSBs after the inhibition of histone deacetylation. Nevertheless, an immediate increase in the levels of RIND-EDSBs was observed when both histone deacetylation and DSB repair were inhibited [4]. These results suggested that RIND-EDSBs are retained and regulated by specific mechanisms that rely on the genome topology and chromatin structures [4].

To search for mechanisms involved in these processes, here we employed the budding yeast *Saccharomyces cerevisiae* as a model system. Because the highly conserved mechanisms that regulate the chromatin structure and DNA repair are well studied in yeast [2], [7], this model organism is advantageous for investigating the roles of various genes in relations to EDSBs. Hence, we modified our assay to measure EDSBs in yeast cells and showed here that yeast genomes similarly possess RIND-EDSBs. To explore the molecular mechanisms regulating RIND-EDSBs, we examined the levels of RIND-EDSBs in a collection of yeast mutants lacking genes in various cellular pathways, including regulators of chromatin structure, endonucleases, and DNA repair. We hypothesized that the level of RIND-EDSBs would be decreased in yeast strains lacking genes involved in RIND-EDSB production or retention, and increased in strains which lack RIND-EDSB repair pathways.

## Materials and Methods

### Yeast strains, media and growth conditions

Yeast strains used in this study are listed in Table 1. Asynchronous yeast cultures were grown in YPD (Sigma, USA) to log phase (OD_600_ 0.4–0.6). For the cell cycle experiments, yeast cells were arrested at G0, G1, S, and M phase by culturing in YP medium containing 2% raffinose (Sigma, USA) for 48 hours, YPD in the presence of 5 µM α-factor (Sigma, USA), of 0.2 M hydroxyurea (Sigma, USA), and of 15 µg/ml nocodazole (Sigma, USA) for 180 minutes at 30°C, respectively. Cell cycle phases were confirmed by phase-contrast microscopy. For the G0 phase, most cells were small and without buds. Cells arrested in the G1 phase had enlarged schmoo morphology. In the early stages of the S phase, cells had large buds and short mitotic spindles. Finally, cells had large buds but no mitotic spindles in the M phase [8]. Apoptosis was induced by the addition of 175 mM acetic acid (pH 3.0) to the YPD for 200 minutes at 30°C [9]. Nine independent preparations of G0 cells from each mutant strain were used in all subsequent experiments to determine the RIND-EDSB levels. In most strains, over 80% of the cells were unbudded. (Proportions of unbudded, small budded and large budded cells of all strains are shown in Table S1). To inhibit the activity of histone deacetylases (HDACs), triplicates of stationary cultures were treated with 10 µM of trichostatin A (TSA; Sigma, USA) for 4 hours [4], [10].

**Table 1:** Yeast strains used in this study.

Yeast strains	Genotype	Source
BY4741	*MATa his3*Δ*1 leu2*Δ*0 met15*Δ*0 ura3*Δ*0*	G.R. Fink
*ybr136w*Δ *(mec1*Δ*)*	*MATa his3*Δ*1 leu2*Δ*0 met15*Δ*0 ura3*Δ*0 mec1*Δ *sml1*Δ	M.C. Keogh [21]
*ybl088c*Δ *(tel1*Δ*)*	*MATa his3*Δ*1 leu2*Δ*0 met15*Δ*0 ura3*Δ*0 tel1*Δ*::KanMX*	Open biosystems
*ymr224c*Δ *(mre11*Δ*)*	*MATa his3*Δ*1 leu2*Δ*0 met15*Δ*0 ura3*Δ*0 mre11*Δ*::KanMX*	Open biosystems
*ymr284w*Δ *(yku70*Δ*)*	*MATa his3*Δ*1 leu2*Δ*0 met15*Δ*0 ura3*Δ*0 yku70*Δ*::KanMX*	Open biosystems
*ymr106c*Δ *(yku80*Δ*)*	*MATa his3*Δ*1 leu2*Δ*0 met15*Δ*0 ura3*Δ*0 yku80*Δ*::KanMX*	Open biosystems
*ylr265c*Δ *(nej1*Δ*)*	*MATa his3*Δ*1 leu2*Δ*0 met15*Δ*0 ura3*Δ*0 nej1*Δ*::KanMX*	Open biosystems
*yer095w*Δ *(rad51*Δ*)*	*MATa his3*Δ*1 leu2*Δ*0 met15*Δ*0 ura3*Δ*0 rad51*Δ*::KanMX*	Open biosystems
*ypr052c*Δ *(nhp6a*Δ*)*	*MATa his3*Δ*1 leu2*Δ*0 met15*Δ*0 ura3*Δ*0 nhp6a*Δ*::KanMX*	Open biosystems
*ybr089c-a*Δ *(nhp6b*Δ*)*	*MATa his3*Δ*1 leu2*Δ*0 met15*Δ*0 ura3*Δ*0 nhp6b*Δ*::KanMX*	Open biosystems
*ydl002c*Δ *(nhp10*Δ*)*	*MATa his3*Δ*1 leu2*Δ*0 met15*Δ*0 ura3*Δ*0 nhp10*Δ*::KanMX*	Open biosystems
*ypr065w*Δ *(rox1*Δ*)*	*MATa his3*Δ*1 leu2*Δ*0 met15*Δ*0 ura3*Δ*0 rox1*Δ*::KanMX*	Open biosystems
*ykl032c*Δ *(ixr1*Δ*)*	*MATa his3*Δ*1 leu2*Δ*0 met15*Δ*0 ura3*Δ*0 ixr1*Δ*::KanMX*	Open biosystems
*ydr174w*Δ *(hmo1*Δ*)*	*MATa his3*Δ*1 leu2*Δ*0 met15*Δ*0 ura3*Δ*0 hmo1*Δ*::KanMX*	Open biosystems
*ymr072w*Δ *(abf2*Δ*)*	*MATa his3*Δ*1 leu2*Δ*0 met15*Δ*0 ura3*Δ*0 abf2*Δ*::KanMX*	Open biosystems
*ycr077c*Δ *(pat1*Δ*)*	*MATa his3*Δ*1 leu2*Δ*0 met15*Δ*0 ura3*Δ*0 pat1*Δ*::KanMX*	Open biosystems
*yol006c*Δ *(top1*Δ*)*	*MATa his3*Δ*1 leu2*Δ*0 met15*Δ*0 ura3*Δ*0 top1*Δ*::KanMX*	Open biosystems
*ylr234w*Δ *(top3*Δ*)*	*MATa his3*Δ*1 leu2*Δ*0 met15*Δ*0 ura3*Δ*0 top3*Δ*::KanMX*	Open biosystems
*ygl175c*Δ *(sae2*Δ*)*	*MATa his3*Δ*1 leu2*Δ*0 met15*Δ*0 ura3*Δ*0 sae2*Δ*::KanMX*	Open biosystems
*ykl113c*Δ *(rad27*Δ*)*	*MATa his3*Δ*1 leu2*Δ*0 met15*Δ*0 ura3*Δ*0 rad27*Δ*::KanMX*	Open biosystems
*ykl114c*Δ *(apn1*Δ*)*	*MATa his3*Δ*1 leu2*Δ*0 met15*Δ*0 ura3*Δ*0 apn1*Δ*::KanMX*	Open biosystems
*yhl022c*Δ (*spo11*Δ*)*	*MATa his3*Δ*1 leu2*Δ*0 met15*Δ*0 ura3*Δ*0 spo11*Δ*::KanMX*	Open biosystems
*ykr101w*Δ *(sir1*Δ*)*	*MATa his3*Δ*1 leu2*Δ*0 met15*Δ*0 ura3*Δ*0 sir1*Δ*::KanMX*	Open biosystems
*ydl042c*Δ *(sir2*Δ*)*	*MATa his3*Δ*1 leu2*Δ*0 met15*Δ*0 ura3*Δ*0 sir2*Δ*::KanMX*	Open biosystems
*ylr442c*Δ *(sir3*Δ*)*	*MATa his3*Δ*1 leu2*Δ*0 met15*Δ*0 ura3*Δ*0 sir3*Δ*::KanMX*	Open biosystems
*ydr227w*Δ *(sir4*Δ*)*	*MATa his3*Δ*1 leu2*Δ*0 met15*Δ*0 ura3*Δ*0 sir4*Δ*::KanMX*	Open biosystems
*ynl330c*Δ *(rpd3*Δ*)*	*MATa his3*Δ*1 leu2*Δ*0 met15*Δ*0 ura3*Δ*0 rpd3*Δ*::KanMX*	Open biosystems
*ynl021w*Δ *(hda1*Δ*)*	*MATa his3*Δ*1 leu2*Δ*0 met15*Δ*0 ura3*Δ*0 hda1*Δ*::KanMX*	Open biosystems
*ydr334w*Δ *(swr1*Δ*)*	*MATa his3*Δ*1 leu2*Δ*0 met15*Δ*0 ura3*ΔΔ*0 swr1*Δ*::KanMX*	Open biosystems
*yol012c*Δ *(htz1*Δ*)*	*MATa his3*Δ*1 leu2*Δ*0 met15*Δ*0 ura3*Δ*0 htz1*Δ*::KanMX*	Open biosystems

### High-Molecular weight (HMW) DNA preparation for yeast and Ty1-EDSB-LMPCR

To prepare HMW DNA, yeast cells were treated with 1 mg/ml lyticase (70 U/mg) (Sigma, USA) for 2 hours and embedded in 1% low melting point agarose at a concentration of 2×10^8^ cells per plug. Embedded cells were digested in 400 µl of digestion buffer (1 mg/ml proteinase K, 50 mM Tris, pH 8.0, 20 mM EDTA, 1% sodium lauryl sarcosine) at 37°C overnight. The plugs were rinsed 6 times in TE buffer for 40 minutes. EDSBs with cohesive ends were polished by incubating with T4 DNA polymerase (New England Biolabs, Beverly, MA, USA) and dNTPs for 1 hour. The enzyme was inactivated by adding EDTA at a final concentration of 20 mM, for 5 minutes, and rinsed 6 times in TE buffer for 40 minutes. The modified LMPCR linkers were prepared from oligonucleotides: 5′-AGGTAACGAGTCAGAC CACCGATCGCTCGGAAGCTTACCTCGTGGACGT-3′ and 5′-ACGTCCACGAG-3′ (Sigma, Singapore) [3]. The linkers (50 pmol) were ligated to the polished EDSB ends in the HMW DNA preparations using T4 DNA ligase (New England Biolabs) at 25°C overnight. Linker-ligated DNA was then extracted from the agarose plugs using a QIAquick gel extraction kit (Qiagen, Basel, Switzerland) [3]. The quantity of EDSBs was measured by real-time PCR using an ABI PRISM® 7500 system (Applied Biosystems, Carlsbad, CA, USA) with Ty1 primer 5′-AATGGAATCCCAACAATTATCTCAA-3′ (Biodesign, Thailand), the linker primer and the Taqman probe homologous to the 3′ linker sequence (6-fam) ACGTCCACGAGGTAAGCTTCCGAGCGA (tamra, phosphate) [Sigma, Singapore] [3]. DNA amplification was performed with 0.2 µM of each primer, 0.3 µM Taqman probe, 0.025 U of HotStarTaq, 1×TaqMan® Universal PCR Master Mix (Applied Biosystems) and 10 ng of ligated DNA. Initial denaturation was at 95°C for 15 minutes, followed by denaturation at 95°C for 5 seconds, annealing at 58°C for 5 seconds, and extension for 2 minutes at 69°C for up to 60 cycles, with quantification after each extension step [3]. To normalize potential differences in the amount of Ty1 per genome, genomic DNA of each mutant was used as its own control DNA. Control DNA was digested with *AluI* and ligated to the LMPCR linkers. The numbers of EDSBs were compared with the *AluI*-digested ligated control DNA and reported in arbitrary units of Ty1-EDSB–LMPCR templates per genome. The Ty1-EDSB-LMPCR units were estimated from the number of *AluI* sites in yeast genome, and converted to the number of EDSBs.

### Yeast nuclei isolation and intranuclear linker ligation

To isolate the nuclei, yeast cells were treated with 1 mg/ml lyticase (70 U/mg) (Sigma, USA) for 2 hours and digested in SPC digestion buffer (1 M sorbitol, 20 mM 1,4-piperazinediethanesulfonic acid (Pipes), pH 6.3, 0.1 mM CaCl_2_), and the nuclei were collected in SPC buffer with 9% ficoll, as previously described [11]. LMPCR linkers were ligated in situ with the nuclei preparations and Ty1-EDSB-LMPCR were performed as described for HMW DNA [3].

### HMGB1si cells and RT-PCR of HMGB1

The commercially available oligonucleotides HSS142453, HSS142454, and HSS142455 from the Stealth RNAi system (Invitrogen) were used for the specific knockdown of HMGB1 gene in HeLa cells. Transfection was carried out with the Lipofectamine2000 transfection reagent (Invitrogen). A negative control siRNA (Invitrogen) was transfected in parallel. After 72 hours, an aliquot of transfected cells was collected to determine the level of HMGB1 mRNA. RNA extraction was performed and 5 µg of RNA was reverse transcribed with RevertAid™ First Strand cDNA Synthesis Kit (Fermentas). The total cDNA of each sample was analyzed in triplicate by a quantitative - comparative CT (ΔΔCT) study in an ABI PRISM® 7500 instrument (Applied Biosystems, Carlsbad, CA, USA) with the HMGB1 forward primer 5′ATATGGCAAAAGCGGACAAG-3′ and the HMGB1 reverse primer 5′GCAACATCACCAATGGACAG-3′ [Sigma, Singapore] [12]. The relative expression of HMGB1 was normalized to GAPDH expression.

### HMW DNA preparation for HMGB1si cells and LINE-1-EDSB-LMPCR

HMW DNA was prepared as previously described [3]. Approximately 5×10^5^ cells were embedded in 1% low melting point agarose, lysed and digested in 400 ml of digestion buffer (1 mg/ml proteinase K, 50 mM Tris, pH 8.0, 20 mM EDTA, 1% sodium lauryl sarcosine) at 37°C overnight. EDSB end polishing and linker ligation were carried out as described for yeast HMW DNA.

For human cells, Long INterspersed Element1 (LINE-1 or L1) sequences were used instead of Ty1 for LMPCR. The number of L1-EDSBs was measured by real-time PCR as previously described [3]. Control HeLa DNA was digested with *EcoRV*/*AluI* and ligated to the LMPCR linkers. The amounts of EDSBs were compared with the *EcoRV/AluI*-digested ligated control DNA and the arbitrary units of L1-EDSB–LMPCR templates per genome were converted to EDSB numbers.

### COmbined Bisulfite Restriction Analysis (COBRA)-LINE-1 and COBRA-LINE-1-EDSB

Nested COBRA-LINE-1 LMPCR was used to measure the methylation of LINE-1 sequences proximal to EDSBs [13]. In the first round, we performed a PCR with 5 µl (approximately 250 ng) of bisulfite-treated DNA, 0.3 µM of AMETLINKP primer (5′-GTTTGGAAGTTTATTTTGTGGAT-3′) and 0.3 µM of LINEMSPCR 270 & 280 reverse primer (5′ RTAAAACCCTCCRAACCAAATA TAAA3′). The PCR conditions were 95°C for 15 minutes, followed by 30 cycles at 95°C for 1 minute, 48°C for 1 minute, and 72°C for 2 minutes and 1 final cycle at 72°C for 7 minutes. In the second round, we performed PCR with 2 µl of PCR amplicons from the first PCR step and 0.3 µM of the L1 primers, the FCOBRALINE-I forward primer (5′ CGTAAGGGGTTAGGGAG TTTTT 3′) and the LINEMSPCR 270 & 280 reverse primers. The PCR conditions were 95°C for 15 minutes, followed by 40 cycles at 95°C for 1 minute, 50°C for 1 minute, and 72°C for 1 minute and 1 final cycle at 72°C for 7 minutes. The restriction analysis was performed by digesting 8 µl of the PCR products with 2 U of *Taq*I and 2 U of *Tas*I at 65°C overnight; the digested products were then electrophoresed in an 8% non-denaturing polyacrylamide gel and stained with SYBR green. DNA methylation levels were determined as previously described [14]. Briefly, the intensities of 4 out of 5 COBRA LINE-1 fragments (the 160, 98, 80 and 62 bp fragments but not the 18 bp fragments) in the polyacrylamide gel were quantified using a phosphoimager and ImageQuant Software (Molecular Dynamics, GE Healthcare, Slough, UK). The intensity of each band was divided by its double-stranded DNA length to normalize the intensity to fragment sizes as follows: 160 bp/160 (A), 98 bp/94 (B), 80 bp/78 (C) and 62 bp/62 (D). The LINE-1 methylation level was calculated by the following formula: (C+A)/(C+A+A+B+D)×100. DNA from HeLa cells was used as a control to normalize the inter-assay methylation variation for all of the experiments.

### Statistical analyses

Statistical analyses were performed using Student's t-test for data with normal distribution or a Mann-Whitney test for data that do not have normal distribution, as specified.

## Results

### Establishment of Ty1-EDSB-LMPCR assay

**Figure 1:**
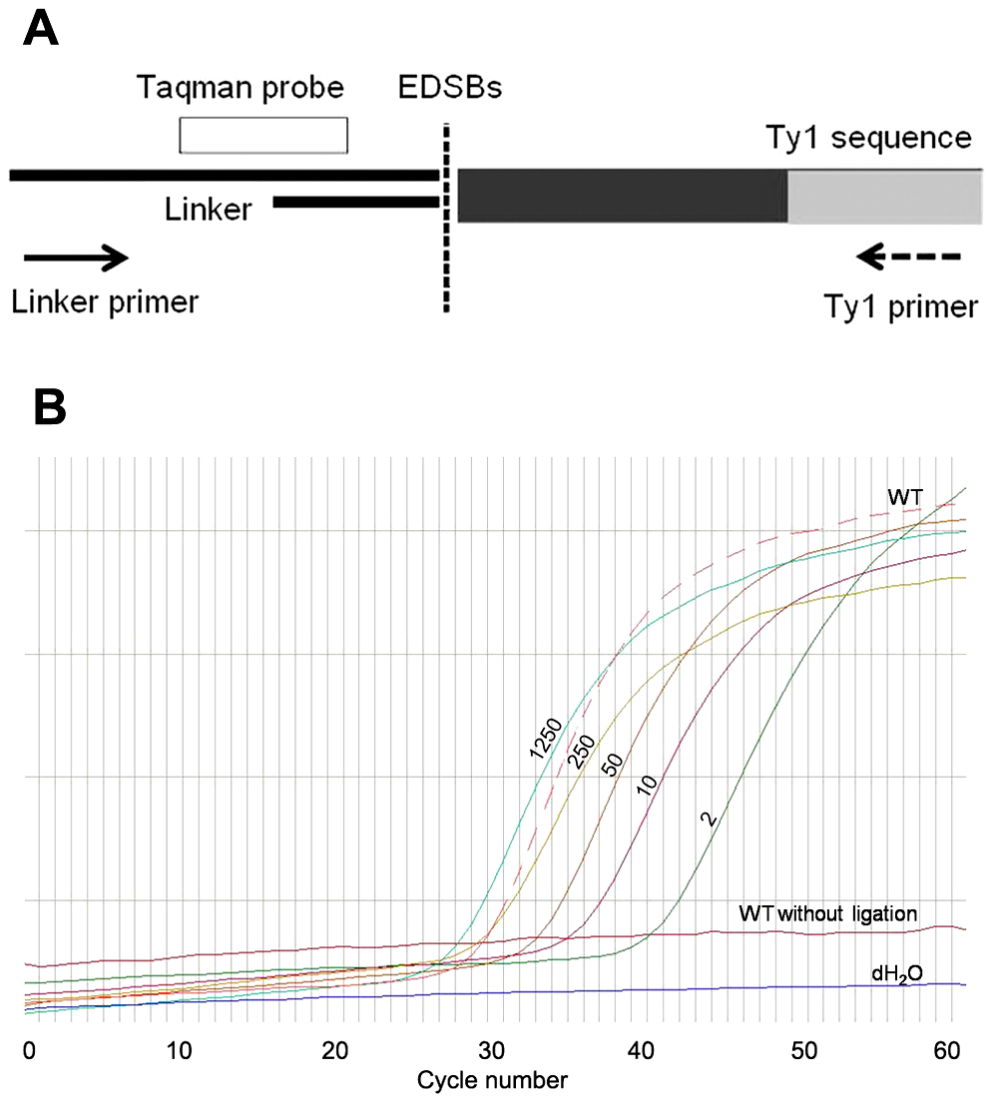
Ty1-EDSB-LMPCR assay. (A) A schematic representation of the Ty1-EDSB-LMPCR assay shows EDSBs in HMW DNA ligated with linker oligonucleotides. EDSBs located close to the Ty1 sequence are measured by real-time PCR using the Ty1 primer (dash arrow), linker primer (solid arrow), and the Taqman probe complementary to the linker sequence (white bar). (B) A representative real-time PCR result shows the level of Ty1-EDSB–LMPCR products in the HMW-DNA of a wild-type yeast strain (WT, dashed line) relative to control DSBs generated by *Alu1* digested restriction enzymes (equivalent to 2, 10, 50, 250 and 1250 cells/µl; dark green, purple, brown, yellow, and light green lines, respectively). Note that no Ty1-EDSB–LMPCR product could be detected in HMW without linker ligation (magenta line) and in water control (dH_2_O, blue line).

We previously established a method to detect EDSBs that occur rather rarely in the human genome. In this assay, genomic DNA was extracted from human cells by the high molecular weight DNA (HMW) preparation protocol [3]. Existing DNA breaks preserved in the genome were first ligated to linker oligonucleotides. They were then detected by PCR using a pair of primers complementary to the linker sequence and the LINE-1 repetitive sequences in human genome. Because this method can detect low numbers of EDSBs occurring in proximity to the LINE-1 sequences, it is called “L1-EDSB-LMPCR” [3]. Here, we modified this method to measure EDSBs in the yeast genome by taking advantage of the Ty1 sequences (as opposed to the LINE-1 sequence in human cells), and called this assay “Ty1-EDSB-LMPCR”. Ty1 sequences are abundant repetitive sequences that intersperse throughout the yeast genome. The presence of EDSBs was quantitatively analyzed by real-time PCR using primers complementary to both the linker and the Ty1 sequences and a Taqman probe complementary to the linker oligonucleotides (Figure 1A). Thus, this assay favourably detected EDSBs located near Ty1 (Ty1-EDSBs). To be efficiently amplified by real-time PCR, EDSBs should locate approximately within 300 bp from IRS sequences, as described for LINE1-EDSBs in human cells [4]. Analyses of varying amounts of control *AluI* digested DNA showed that our assay quantitatively detected DSB ends and did not detect any Ty1-EDSB-LMPCR product without linker ligation (Figure 1B).

### Detection of EDSBs in yeast

**Figure 2:**
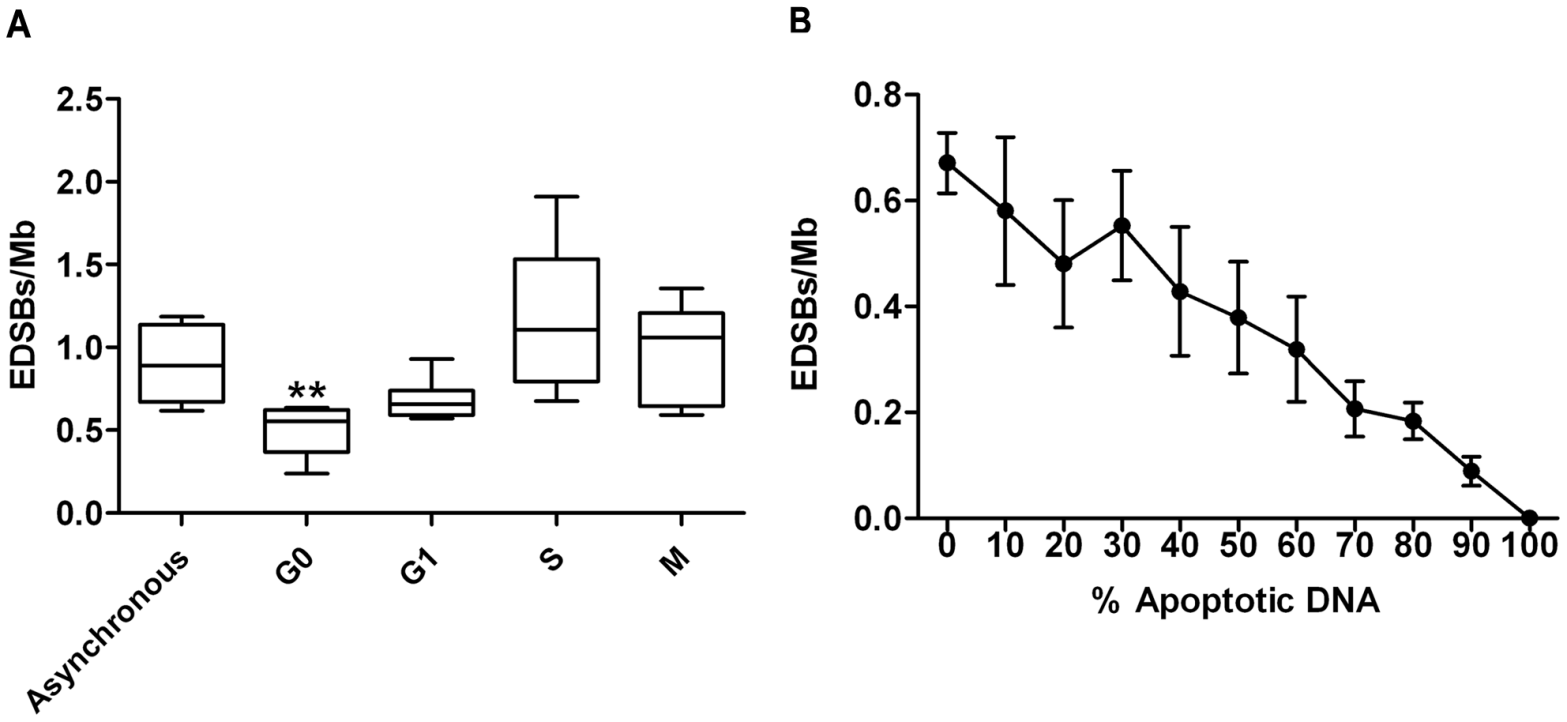
EDSBs in various phases of the cell cycle. (A) EDSBs were measured in asynchronous culture and in yeast cells arrested in G0, G1, S, and M phases. The levels of EDSBs from 9 independent experiments are shown as box plots, with the boxes representing the interquartile ranges (25^th^ to 75^th^ percentile) and the median lines representing the 50^th^ percentile. The whiskers represent the minimum and the maximum values. There was a significant decrease in EDSBs in G0 cells compared to asynchronous culture, such that **P<0.001 (Mann-Whitney test). (B) HMW DNA was isolated from apoptotic yeast cells, mixed with control DNA at varying percentages, and analyzed by Ty1-EDSB-LMPCR. The graph represents the mean levels of EDSBs with error bars representing standard deviations. The Ty1-EDSB-LMPCR assay could not detect DNA fragments from apoptotic cells (at 100% apoptotic DNA). Furthermore, apoptotic DNA fragments did not interfere with quantitative measurement of EDSBs.

We used the Ty1-EDSB-LMPCR assay to estimate the levels of EDSBs during the G0, G1, S and M phases of the cell cycle (Figure 2A). Here, we estimated the total amount of EDSBs under an assumption that the *AluI* restriction endonuclease generates DSBs every 256 bp on average, and that EDSBs are distributed equally throughout the genome. The results were similar to the findings in human cells; the level of EDSBs was highest in S phase, but still detectable at a lower level in G0 phase (Figure 2A). Next, we tried to verify, if the fragmented DNA from apoptotic cells could interfere with the detection of the genuine EDSBs. Therefore, we tested whether Ty1-EDSB-LMPCR could detect fragmented DNA generated by apoptosis. Yeast cells undergo apoptotic cell death, with characteristic DNA fragmentation, upon treatment with acetic acid [9]. We found that Ty1-EDSB-LMPCR did not detect fragmented apoptotic DNA, prepared by the HMW DNA extraction protocol (Figure 2B). This is likely due to the nature of the DSB ends of apoptotic DNA fragments which was reported to be staggered, not efficiently blunted by Klenow treatment, and thus are not ligated to the linkers [9]. Ty1-EDSB-LMPCR specifically detected only EDSBs from the genomic DNA, which was added to the sample to normalize the total amount of DNA, and did not detect any signal from the sample containing 100% fragmented DNA from apoptotic cells (Figure 2B, at 100% Apoptotic DNA). This indicated that apoptotic DNA did not interfere with the Ty1-EDSB-LMPCR measurement of EDSBs. Therefore, the Ty1-EDSB-LMPCR is an accurate and sensitive method to study EDSBs and RIND-EDSBs in the yeast genome.

**Figure 3:**
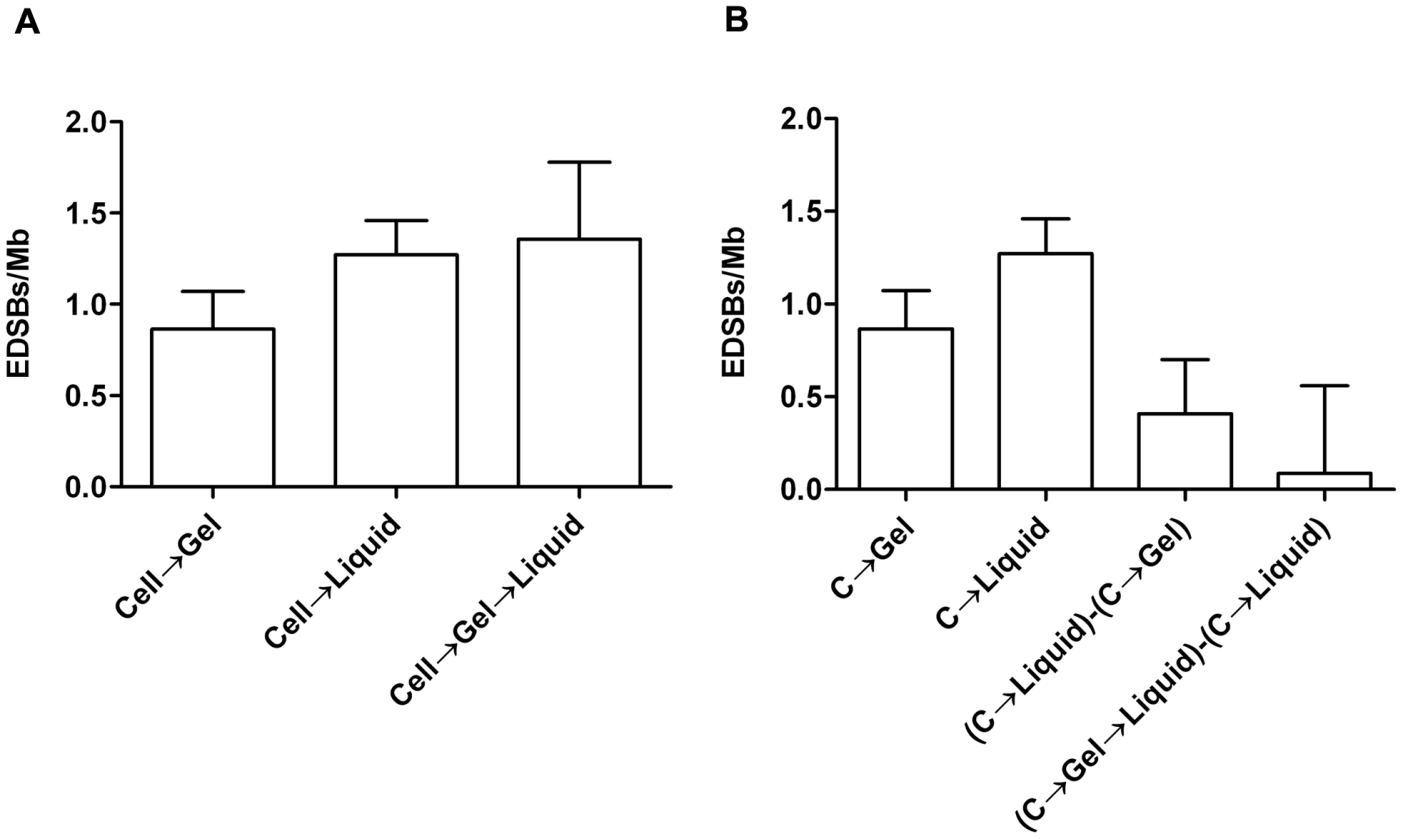
EDSBs were detected in different DNA preparations including HMW DNA (cell→gel), liquid DNA (cell→liquid), and liquid DNA extracted from in-gel HMW DNA (cell→gel→liquid). (A) The levels of EDSBs from different DNA preparation methods. (B) Subtracted DSBs levels between liquid DNA and other methods. When comparing cell→gel→liquid with cell→liquid, adding in gel preparation step did not increase the number of DSBs significantly. The average levels of EDSBs from 9 independent experiments are shown as histograms with error bars representing SEM.

We also determined if the signals we observed by Ty1-EDSB-LMPCR were due to our DNA preparation protocol. Similarly to the experiments performed in human cells [3], we compared the levels of EDSBs of genomic DNA prepared with different protocols, including an in-gel (HMW-DNA), a liquid DNA, and a combined preparation protocols (Figure 3). Only a minimal difference was derived when we subtracted the DSBs levels of DNA prepared with the liquid DNA protocol (cell→liquid) from that of DNA prepared with the combined in-gel followed by liquid DNA protocol (cell→gel→liquid) (Figure 3B). This suggests that adding an in-gel preparation step did not increase the number of DSBs significantly. Thus, our method for in-gel preparation of genomic DNA produced an insignificant number of breaks, as measured by Ty1-EDSB-LMPCR.

**Figure 4:**
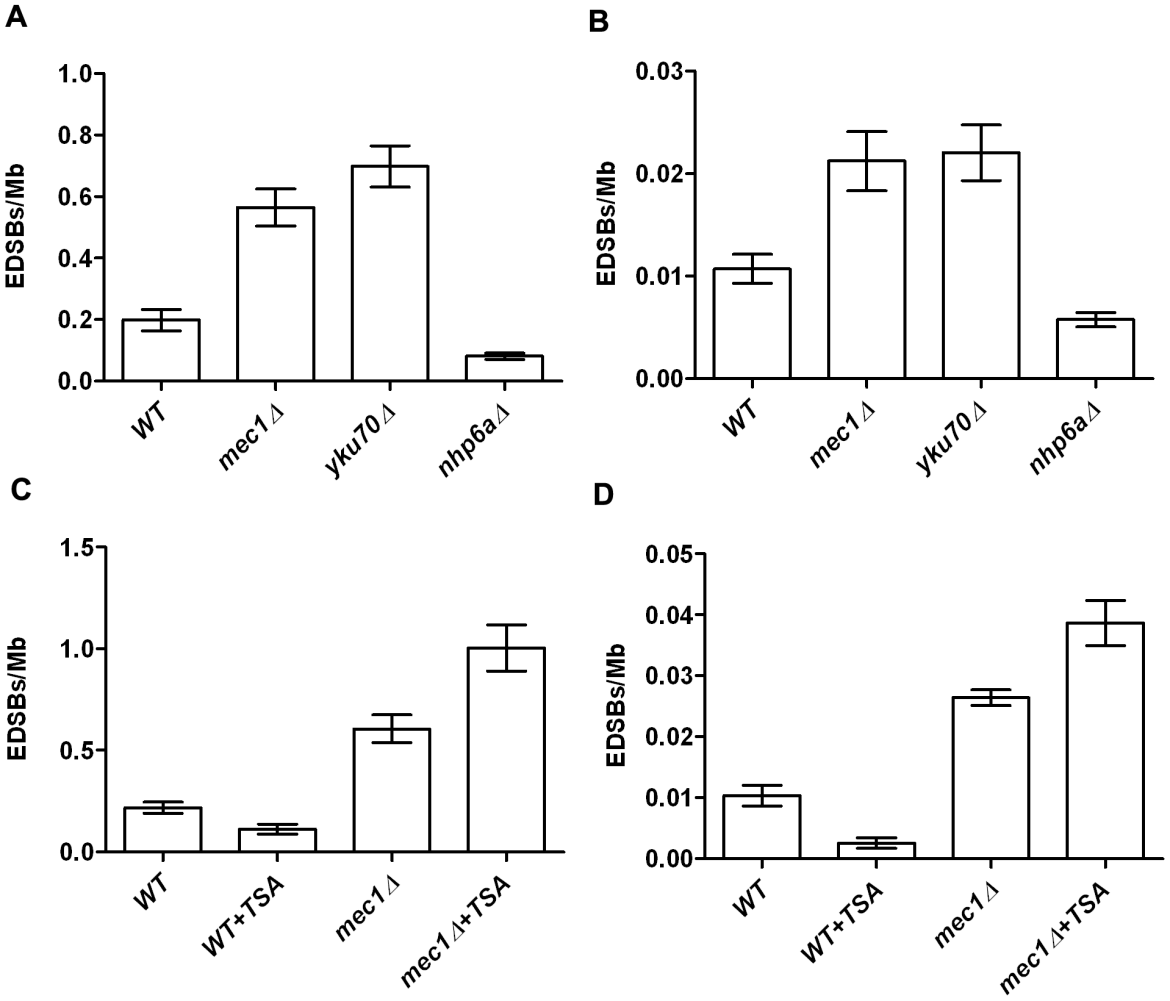
RIND-EDSBs levels using HMW DNA preparation and intranuclear ligation protocols. (A, B) The levels of RIND-EDSBs were measured in G0 cells of WT, *mec1*Δ, *yku70*Δ, *nhp6a*Δ strains using HMW DNA (A) and intranuclear ligation (B) protocols. (C, D) The levels of RIND-EDSBs in controls and TSA-treated WT and *mec1*Δ strains using HMW DNA (C) and intranuclear ligation (D) protocols. Bar graphs represent average values and error bars represent standard deviation of triplicate experiments.

To further confirm that Ty-EDSBs are not artifacts from HMW DNA preparation, we isolated the yeast nuclei and performed linker ligation *in situ*. Using this intranuclear ligation method, the DNA was protected in the nuclear membrane. We compared the levels of Ty-EDSBs of various mutant yeast strains that harbor different levels of Ty-EDSBs (see below) using the HMW DNA preparation and the intranuclear ligation protocols (Figure 4A and B, respectively). The results demonstrated that the assay could detect DNA breaks within the nuclei. Importantly, we observed the same pattern of differences in the levels of EDSBs among different yeast mutant strains using the 2 techniques, suggesting that the HMW-DNA-based assay could reflect the situations in the nuclei. Although the levels of EDSB detected with intranuclear ligation were lower than those detected with the HMW DNA preparations, this could be expected from the lower efficiency of the ligation reaction in the complex nuclear architecture. It is also unlikely that the mutations in various genes would lead to the same effect in these 2 different protocols if they affect artificially induced DNA breaks. Therefore, we believe that the assay provide a sensitive means to measure the low level of randomly occurring EDSBs that reflect the levels *in vivo*.

In our previous study in human cells, when we treated the cells with a histone deacetylase inhibitor trichostatin A (TSA), histones became hyperacetylated and the retained RIND-EDSBs were repaired [4]. However, the levels of RIND-EDSBs increased when cells were treated simultaneously with TSA and inhibitors of DNA-PKcs and ATM [4]. Intriguingly, similar results were observed in yeast cells suggesting that RIND-EDSBs were also similarly regulated by chromatin structure in yeast (Figure 4C). The levels of RIND-EDSBs were decreased in TSA treated cells and increased in TSA-treated *mec1Δ* cells lacking the ATR checkpoint kinase homolog. In this experiment, we demonstrated again that both HMW DNA preparation and intranuclear ligation protocols yielded the same results (Figure 4C and 4D).

### Repair of RIND-EDSBs

**Figure 5:**
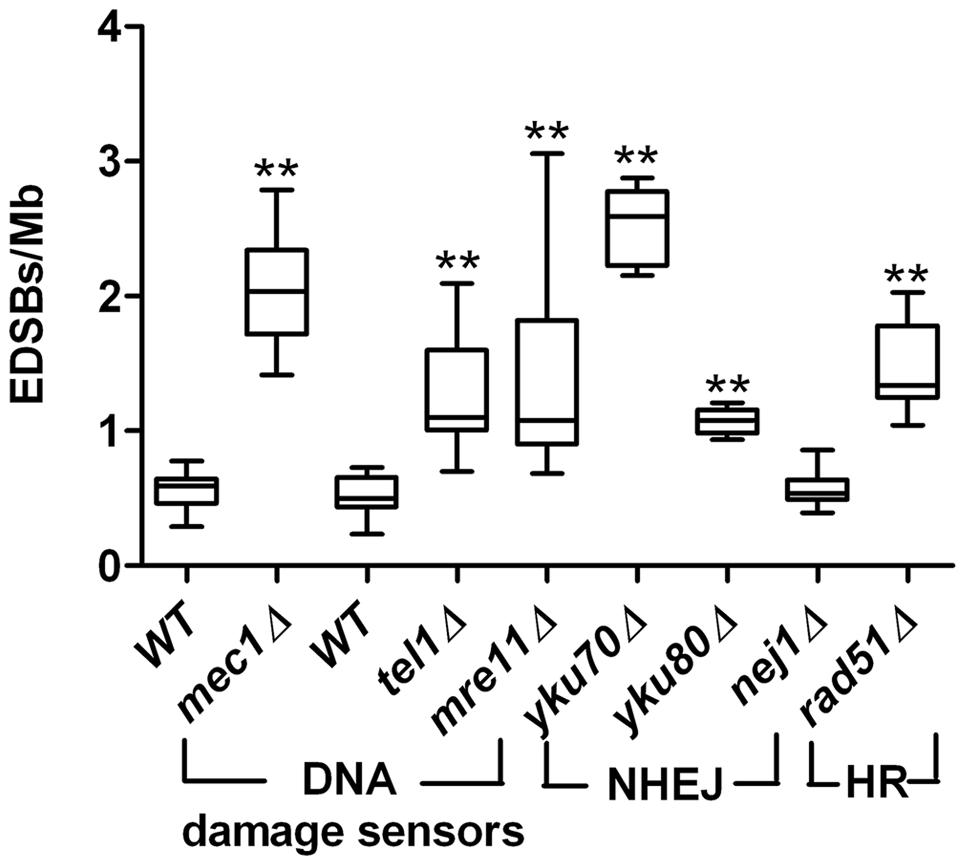
Levels of RIND-EDSBs in yeast strains with mutations in DSB repair pathways. The levels of RIND-EDSBs were significantly increased in G0 cells of *mec1*Δ, *tel1*Δ, *mre11*Δ, *yku70*Δ, *yku80*Δ, and *rad51*Δ but not in *nej1*Δ strains. The levels of EDSBs from 9 independent experiments are shown as box plots, with the boxes representing the interquartile ranges (25^th^ to 75^th^ percentile) and the median lines representing the 50^th^ percentile. The whiskers represent the minimum and the maximum values. **P<0.001 (Mann-Whitney test).

The sensitivity of our PCR-based DSB detection method allowed us to quantitatively analyze low levels of EDSBs in non-replicative (G0) cells. To explore the roles of DNA repair pathways in RIND-EDSB repair, we examined the levels of RIND-EDSBs in several yeast strains with deletions of genes encoding components of the DNA damage response (Figure 5). The levels of RIND-EDSBs in G0 yeast cells were significantly increased in the *mec1*Δ, *tel1*Δ, and *mre11*Δ strains, which lack key DNA damage sensor genes. We then examined the levels of RIND-EDSBs in strains lacking genes important for NHEJ repair (*yku70*Δ, *yku80*Δ, and *nej1*Δ). The levels of RIND-EDSBs were significantly increased in strains *yku70*Δ and *yku80*Δ. However, when *NEJ1* was deleted, there was no change in the RIND-EDSB level. During G0 phase of haploid yeast cells, we did not expect that the RIND-EDSBs would be repaired by homologous-recombination (HR). However, we observed a large increase in RIND-EDSBs in a yeast strain lacking Rad51, a key protein in the HR pathway.

### Regulations of RIND-EDSBs by chromosomal stress, and endonucleases

Whether there is any cellular process that influences RIND-EDSB levels is still not known. Without DNA replication, the RIND-EDSBs could be a result of chromosomal stress or an endonuclease. We focused on 3 groups of genes whose functions promote DNA breaks, including topoisomerases, endonucleases and High-Mobility Group B (HMGB). We hypothesized that RIND-EDSBs would be lower in yeast strains lacking any genes involved in the production or the retention of RIND-EDSBs.

**Figure 6:**
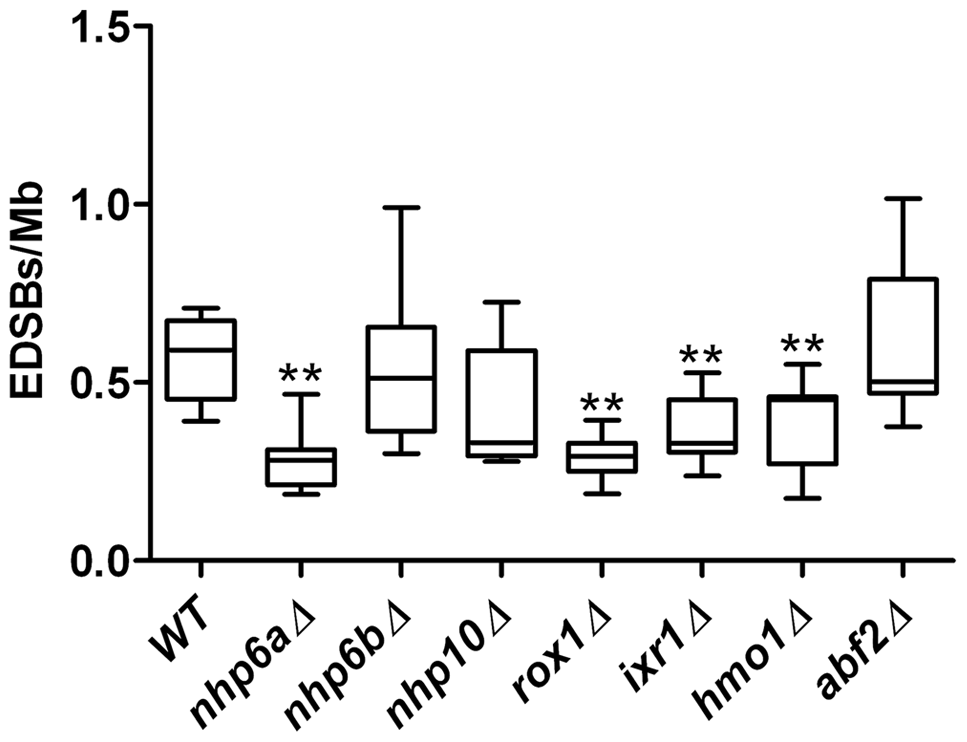
Levels of RIND-EDSBs in yeast strains with deletions of genes encoding proteins with the High-Mobility Group B (HMGB) domain. The levels of EDSBs were significantly decreased in G0 cells of yeast strains lacking *NHP6A*, *IXR1*, *ROX1*, and *HMO1*, suggesting that they play an important role in the production or retention of RIND-EDSBs. Nevertheless, the levels of RIND-EDSBs in *nhp6b*Δ, *nhp10*Δ, and *abf2*Δ strains were unchanged. The levels of EDSBs from 9 independent experiments are shown as box plots, with the boxes representing the interquartile ranges (25^th^ to 75^th^ percentile) and the median lines representing the 50^th^ percentile. The whiskers represent the minimum and the maximum values. **P<0.001 (Mann-Whitney test).

**Figure 7:**
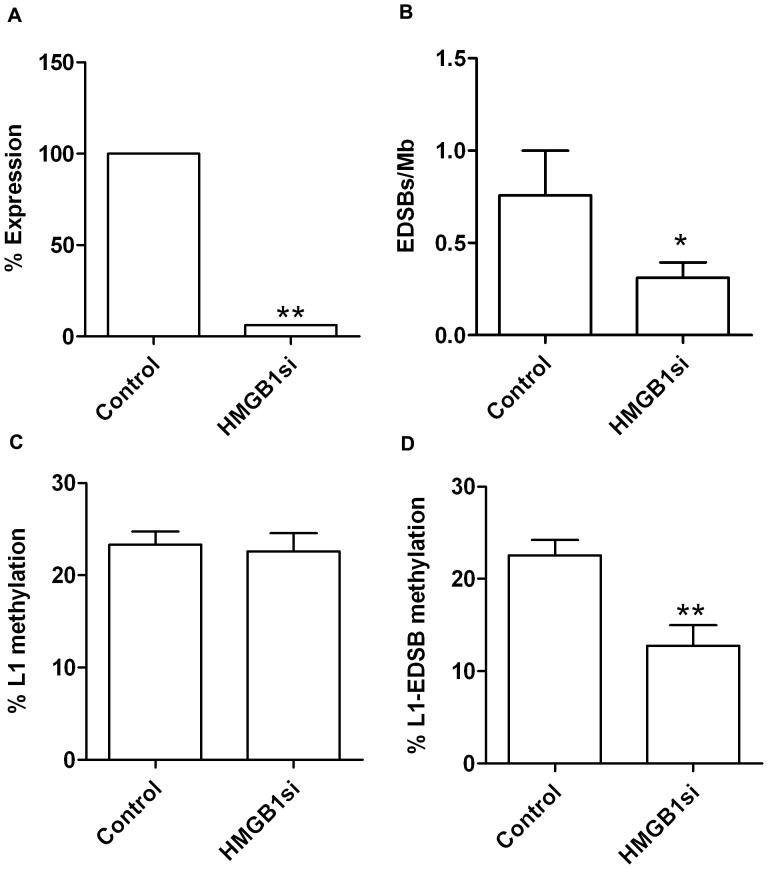
Levels of RIND-EDSBs in HeLa cells transfected with HMGB1 siRNA. (A) The level of expression of HMGB1 mRNA was significantly downregulated in HMGB1 siRNA HeLa cells compared to control siRNA. **P<0.001 (Paired t-test). (B) Downregulation of HMGB1 by HMGB1 siRNA in HeLa cells resulted in a decreased level of RIND-EDSBs when compared to the control. (C) Levels of L1 methylation as measured by COBRA-L1 assay were not changed in HMGB1 siRNA transfected cells. (D) L1–EDSB methylation levels were significantly lower in HMGB1 siRNA cells than in the control. The mean values from 9 independent experiments are shown as histograms with error bars representing SEM. *P<0.05, **P<0.001 (t-test).

First, we examined the role of genes encoding proteins with HMGB domains. We measured the levels of RIND-EDSBs in yeast strains with deletions of each of the seven genes in the HMGB family, i.e., *NHP6A, NHP6B, NHP10, ROX1, IXR1, HMO1*, and *ABF2* [15]. The levels of RIND-EDSBs were reduced in all of the mutant strains tested, but significantly decreased in the *nhp6a*Δ, *rox1*Δ, *ixr1*Δ, and *hmo1*Δ strains (Figure 6). This finding led us to investigate the role of HMGB1, the human homolog of budding yeast *HMO1*, in human cells. Indeed, siRNA depletion of HMGB1 in human cervical carcinoma HeLa cells also resulted in a decrease in the level of RIND-EDSBs (Figure 7B).

Our previous study showed that RIND-EDSBs are preferentially retained in methylated DNA [3]. We therefore examined the methylation of genomic LINE1 (Figure 7C) and of LINE1 located close to EDSBs in HeLa cells (Figure 7D). Intriguingly, the depletion of HMGB1 significantly decreased the methylation level of L1-EDSBs (Figure 7D). This result indicates that HMGB1 is involved in the production or retention of hypermethylated RIND-EDSBs in HeLa cells.

**Figure 8:**
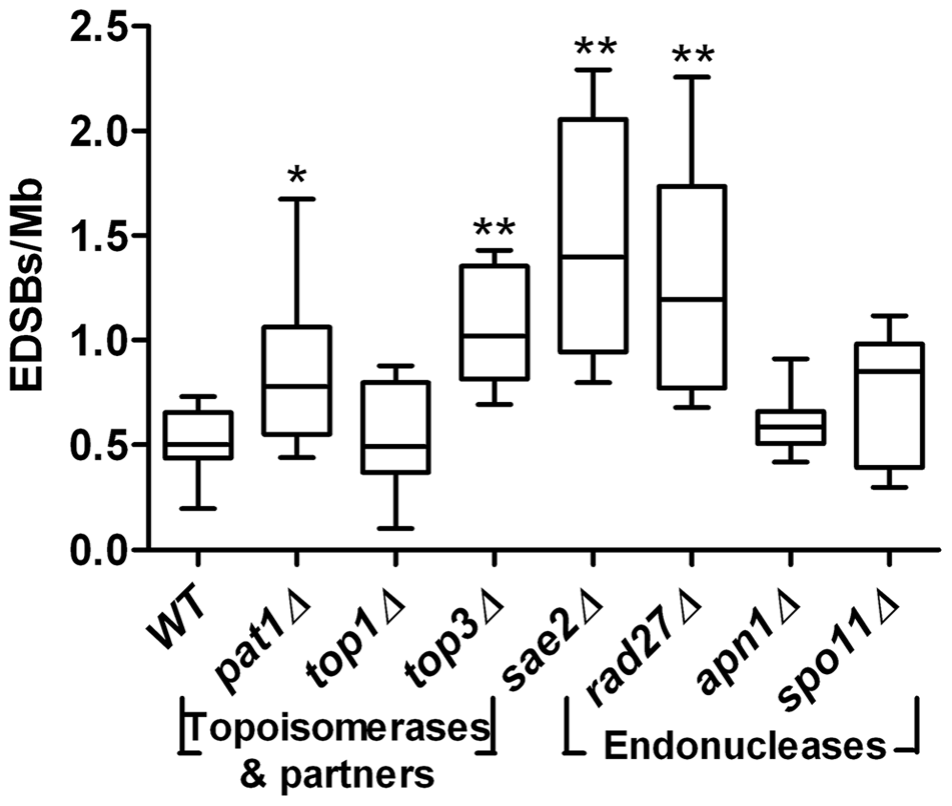
RIND-EDSBs in strains with mutations in genes encoding topoisomerases, their partners, and endonucleases. Deletions of genes encoding topoisomerases, their partners, and endonucleases did not reduce the levels of RIND-EDSBs in G0 cells. On the contrary, the levels of RIND-EDSBs were increased in G0 cells of *top3*Δ, *rad27*Δ, and *sae2*Δ strains. The values from 9 independent experiments are shown as box plots, with the boxes representing the interquartile ranges (25^th^ to 75^th^ percentile) and the median lines representing the 50^th^ percentile. The whiskers represent the minimum and the maximum values. *P<0.05, **P<0.001(Mann-Whitney test).

Next, we examined a potential role of topoisomerases, their partners, and endonucleases in the generation of EDSBs. We found that, while the levels of RIND-EDSBs did not change in the *top1*Δ, *apn1*Δ, and *spo11*Δ strains, they significantly increased in the *pat1*Δ, *top3*Δ, *sae2*Δ, and *rad27*Δ strains (Figure 8).

### Heterochromatin and RIND-EDSBs levels

**Figure 9:**
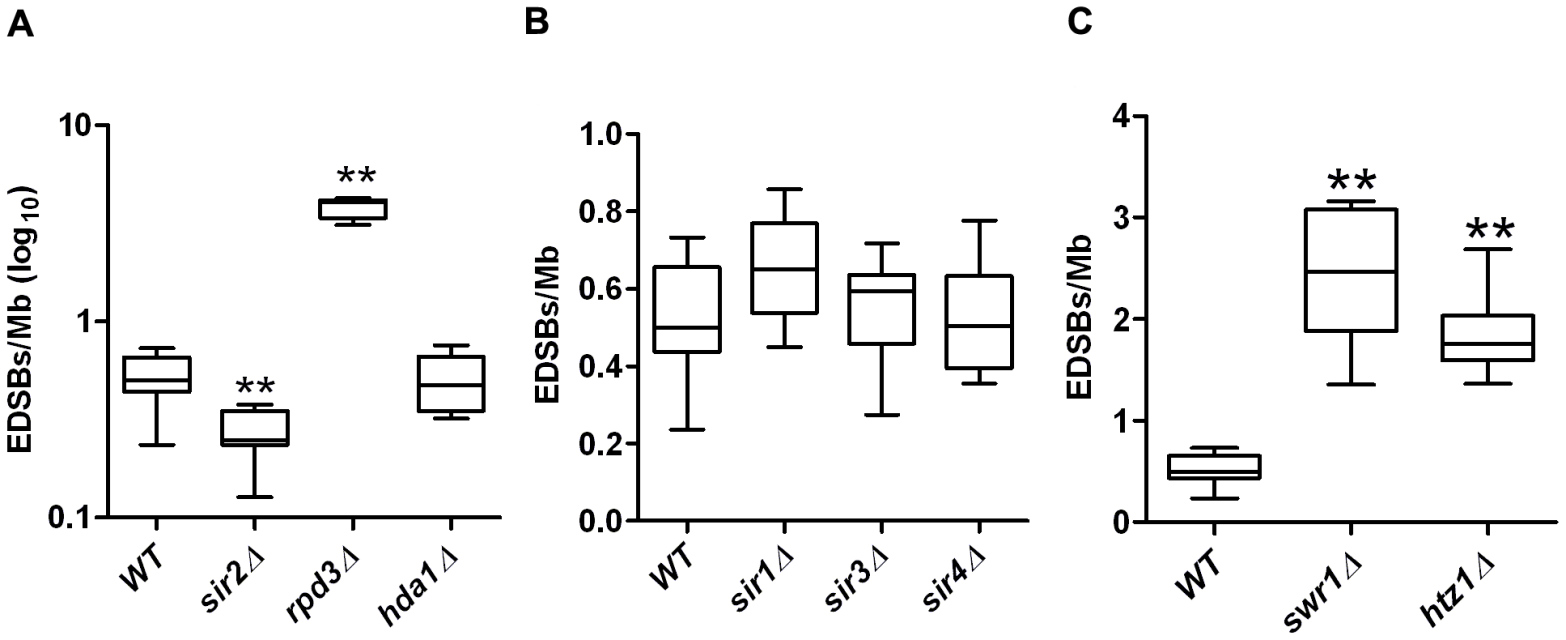
RIND-EDSBs and chromatin regulators. (A) The levels of RIND-EDSBs were measured in G0 cells of yeast strains lacking histone deacetylase genes, *SIR2, RPD3*, and *HDA1*. The level was decreased in the *sir2*Δ strain, while it was increased in *rpd3*Δ strain. (B) No significant change in the level of RIND-EDSBs was observed in yeast strains lacking the silent information regulator genes, *SIR1*, *SIR3*, or *SIR4*. (C) In contrast, deletions of *HTZ1* and *SWR1*, genes required for the prevention of heterochromatin spreading, led to significantly increased levels of RIND EDSBs. The values from 9 independent experiments are shown as box plots, with the boxes representing the interquartile ranges (25^th^ to 75^th^ percentile) and the median lines representing the 50^th^ percentile. The whiskers represent the minimum and the maximum values. **P<0.001 (Mann-Whitney test).

Our previous study demonstrated a relationship between RIND-EDSBs and chromatin acetylation [4]. To determine if these hold true in yeast, we examined the levels of RIND-EDSBs in yeast strains lacking genes important for heterochromatin formation, including the two histone deacetylases *SIR2* and *HDA1*. We observed a significantly lower level of RIND-EDSBs in *sir2*Δ, as predicted, but not in the *hda1*Δ (Figure 9A). We also examined the level of RIND-EDSBs in a mutant strain lacking *RPD3*, a distinct group of histone deacetylase. Unlike *sir2*Δ, we found a significant increase of RIND-EDSB levels in the *rpd3*Δ strain (Figure 9A).

The fact that we detected a lower level of RIND-EDSBs in the *sir2*Δ strain, together with the well-described role of Sir2 in heterochromatin formation, led us to propose that the levels of RIND-EDSBs could be indirectly controlled by Sir2 via its role in heterochromatin formation. Of note, we did not see any significant decrease in the levels of RIND-EDSBs in yeast strains lacking Sir1, Sir3, and Sir4, proteins that are related to Sir2 [16], suggesting that Sir2 may hold a specific role in the regulation of RIND-EDSBs (Figure 9B). On the other hand, when genes that suppress heterochromatin spreading, such as *HTZ1 and SWR1* [17], were deleted, the levels of RIND-EDSBs increased significantly (*htz1*Δ and *swr1*Δ, Figure 9C).

## Discussion

In this study, we established an assay for the measurement of RIND-EDSBs in budding yeast, and investigated the roles of various genes involved in the regulation of chromatin and DNA repair on the levels of RIND-EDSBs.

### RIND-EDSBs are evolutionarily conserved

Similar to what previously observed in human cells [3], we found that EDSBs were present in the yeast genome during all phases of the cell cycle (Figure 2A). The detection of Ty1-EDSBs during the G0 phase indicates that the non-dividing yeast cells harbored RIND-EDSBs. These RIND-EDSBs may be produced in non-replicating cells or possibly during prior cell cycle transition. Additionally, the detection of high levels of S-phase EDSB was consistent with the hypothesis that DNA replication converts single-strand lesions to replication-dependent EDSBs [1]. The similarities between EDSB patterns observed in the mammalian and yeast genomes imply that an existence of EDSBs, and perhaps RIND-EDSBs, are evolutionarily conserved and may be required for cell homeostasis.

We have shown here and previously that, in both human and yeast cells, the levels of RIND-EDSB are actively regulated. Though evolutionarily distant, both species retain mechanisms, more specifically genes, which regulate the baseline levels of the RIND-EDSBs. HMGB domain proteins are abundant proteins that bind DNA in a sequence-independent manner [18]. They are intricately involved in the regulation of chromatin structure and affect many DNA metabolic processes. HMGB proteins bind to certain DNA lesions and either inhibit or facilitate their removal [15]. Interestingly, the levels of RIND-EDSBs were reduced in cells lacking HMGB proteins (Figure 6 and 7). Therefore, the HMGB proteins may have a positive role in retaining high levels of RIND-EDSBs in the yeast genome. The HMGB genes are known to possess diverse functions in the maintenance of chromatin structures. The variation in the degrees of RIND-EDSB reduction in different deletion strains may be a result of functional redundancies within the HMGB family. Nhp6A and Nhp6B are homolog proteins that are functionally redundant. In budding yeast, *NHP6A* is expressed much more robustly than *NHP6B*. Therefore, the deletion of *NHP6A* resulted in a more pronounced phenotype [15].

We observed here that yeast strains lacking specific genes involving in the generation of DNA breaks, topoisomerases and endonucleases, increased RIND-EDSB levels (Figure 8). These results appear contradictory to our initial hypothesis and suggest that these genes are not involved in the production or retention of RIND-EDSBs. The increase in RIND-EDSB levels when the genes encoding certain topoisomerases or endonucleases were deleted may provide a hint for a potentially essential role of RIND-EDSBs. Both topoisomerases and endonucleases generate DNA breaks to mediate their biological roles, which is to release genome's physical stress. Thus, an increase of RIND-EDSBs upon the deletions of these genes may represent a crucial compensatory mechanism for the loss of function of certain topoisomerases or endonucleases. The conserved existence of RIND-EDSBs over long evolutionary time implies that they may provide an advantage for the organisms such that this feature survives through natural selection.

### Levels of RIND-EDSB and the repair of the breaks are regulated

In human cells, compact heterochromatin-associated RIND-EDSBs are repaired by an ATM-dependent pathway. However, Ku-mediated NHEJ can repair euchromatin-associated EDSBs [4]. Here we found that the levels of RIND-EDSBs were significantly increased in yeast strains with deletions of genes encoding components of the DNA damage response (Figure 5). This result suggested that the levels of RIND-EDSBs are constantly monitored and controlled by these DNA damage sensors. Thus, deletion of the DNA damage sensors abolished that control mechanism.

Two major pathways that repair double stranded breaks are non-homologous recombination (NHEJ) and homologous-mediated recombination (HR) [2]. In this study, we focused on a set of genes that operate in the NHEJ pathway. During the non-replicating phase, the haploid budding yeasts contain only one copy of the genome. Thus, it is generally presumed that the conventional HR-mediated DNA repair, which requires another copy of the genome as a template for repair, does not operate during this stage. The levels of RIND-EDSBs were significantly increased in *yku70*Δ and *yku80*Δ strains. Therefore, the NHEJ pathway regulated, at least partly, the levels of RIND-EDSBs in non-replicating yeast. These data are in accordance with our previous finding that RIND-EDSBs could be repaired by the NHEJ pathways in human cells [4]. Interestingly, *nej1* deletion did not change the RIND-EDSB level. There may be other factors that compensate for the loss of *NEJ1* in yeast. Although it is generally believed that in the non-replicative stage, haploid yeasts are not able to repair DNA breaks by the conventional HR-mediated pathway, we observed a significant increase in RIND-EDSBs in a yeast strain lacking Rad51, a key protein in the HR pathway. This result suggests that there might be an alternative Rad51-mediated pathway to repair RIND-EDSBs in non-replicative yeast.

### RIND-EDSBs and heterochromatin

We previously demonstrated, in human cells, that areas containing RIND-EDSBs are hypermethylated and are within the facultative heterochromatin [4]. We also found that the RIND-EDSBs were devoid of γH2AX, and that trichostatin A (TSA) treatment increased histone acetylation, produced spontaneous DNA breakages, triggered H2AX phosphorylation and allowed RIND-EDSB repair [4]. Therefore, we hypothesized that RIND-EDSB levels and break repair are at least partly regulated by specific pathways and are influenced by the genome topology and chromatin structures.

Studies in yeast indicated that the level of RIND-EDSBs is connected to the level of heterochromatin and may be controlled indirectly by the proteins that regulate the spreading of heterochromatin (Figure 9). Our findings were also consistent with our prior report, which suggested that RIND-EDSBs are likely retained in heterochromatin. Low levels of RIND-EDSBs were found in yeast strain lacking the histone deacetylase Sir2. Moreover, *htz1*Δ and *swr1*Δ strains, lacking genes that suppress heterochromatin spreading [17], possessed high levels of RIND-EDSBs. Nevertheless, a significant increase in RIND-EDSB levels in the *rpd3*Δ strain was observed. Rpd3 is a histone deacetylase that has a controversial role in heterochromatin formation. Traditionally, Rpd3 has been associated with telomere stability [19]. However, recent evidence suggests that it may antagonize Sir2-dependent heterochromatin spreading [20]. This result supports the anti-Sir2 role of Rpd3.

## Conclusion

In this study, we devised a novel assay, Ty1-EDSB-LMPCR, and showed that *Saccharomyces cerevisiae*, like human cells, possesses RIND-EDSBs. We measured the levels of RIND-EDSBs in yeast and explored the roles of many genes in the regulation of RIND-EDSBs. By studying yeast strains lacking several genes involved in DNA repair, we found that RIND-EDSBs are repaired by the NHEJ and Rad51-dependent pathways. Furthermore, our results showed that a role of certain genes involving in heterochromatin dynamics or DNA metabolisms is to maintain a level of RIND-EDSBs in the cell. We showed that at least some mechanisms that regulate RIND-EDSB levels are evolutionarily conserved between yeast and human. Interestingly, the lack of proteins that maintain genomic integrity by generating temporary DSBs, such as topoisomerases, increased RIND-EDSBs. This provides a clue to a potential role of RIND-EDSBs in an important physiological function. It is thus interesting to further investigate if there is an advantage for cells to maintain a certain level of RIND-EDSBs.

## Supporting Information

Table S1 **Percentage of unbudded, small budded and large budded yeast cells in G0 cultures.**
(DOC)
