# Supplementary material for: Replication-Independent Endogenous DNA Double-Strand Breaks in Saccharomyces cerevisiae Model
Source: PLoS One. 2013 Aug 19;8(8):e72706. doi: 10.1371/journal.pone.0072706 (PMC3747138; doi:10.1371/journal.pone.0072706)
Supplement: Table S1 — Percentage of unbudded, small budded and large budded yeast cells in G0 cultures. (DOC) [file pone.0072706.s001.doc]

**Table S1. Percentage of unbudded, small budded, and large budded yeast cells in G0 cultures.**

| **Yeast strains** | **% unbudded cells** | **% small budded cells** | **% large budded cells** |
| --- | --- | --- | --- |
| BY4741 | 91 | 9 | 0 |
| *ybr136w∆ (mec1∆)* | 86 | 14 | 0 |
| *ybl088c∆ (tel1∆)* | 85 | 15 | 0 |
| *ymr224c∆ (mre11∆)* | 88 | 12 | 0 |
| *ymr284w∆ (yku70∆)* | 95 | 5 | 0 |
| *ymr106c∆ (yku80∆)* | 85 | 15 | 0 |
| *ylr265c∆ (nej1∆)* | 90 | 10 | 0 |
| *yer095w∆ (rad51∆)* | 85 | 15 | 0 |
| *ypr052c∆ (nhp6a∆)* | 93 | 7 | 0 |
| *ybr089c-a∆ (nhp6b∆)* | 82 | 18 | 0 |
| *ydl002c∆ (nhp10∆)* | 89 | 11 | 0 |
| *ypr065w∆ (rox1∆)* | 84 | 16 | 0 |
| *ykl032c∆ (ixr1∆)* | 92 | 8 | 0 |
| *ydr174w∆ (hmo1∆)* | 86 | 14 | 0 |
| *ymr072w∆ (abf2∆)* | 89 | 11 | 0 |
| *ycr077c∆ (pat1∆)* | 94 | 6 | 0 |
| *yol006c∆ (top1∆)* | 93 | 7 | 0 |
| *ylr234w∆ (top3∆)* | 87 | 13 | 0 |
| *ygl175c∆ (sae2∆)* | 83 | 17 | 0 |
| *ykl113c∆ (rad27∆)* | 91 | 9 | 0 |
| *ykl114c∆ (apn1∆)* | 77 | 13 | 0 |
| *yhl022c∆ (spo11∆)* | 85 | 15 | 0 |
| *ykr101w∆ (sir1∆)* | 78 | 22 | 0 |
| *ydl042c∆ (sir2∆)* | 75 | 25 | 0 |
| *ylr442c∆ (sir3∆)* | 85 | 15 | 0 |
| *ydr227w∆ (sir4∆)* | 74 | 26 | 0 |
| *ynl330c∆ (rpd3∆)* | 82 | 18 | 0 |
| *ynl021w∆ (hda1∆)* | 73 | 27 | 0 |
| *ydr334w∆ (swr1∆)* | 92 | 8 | 0 |
| *yol012c∆ (htz1∆)* | 86 | 14 | 0 |
